# Influencing Factors of Public Satisfaction with COVID-19 Prevention Services Based on Structural Equation Modeling (SEM): A Study of Nanjing, China

**DOI:** 10.3390/ijerph182413281

**Published:** 2021-12-16

**Authors:** Wei Chen, Yijun Shi, Liwen Fan, Lijun Huang, Jingyi Gao

**Affiliations:** 1 School of Geographic and Biologic Information, Nanjing University of Posts and Telecommunications, Nanjing 210023, China; flwcxwz@163.com; 2 School of Landscape Architecture, Zhejiang A&F University, Hangzhou 311300, China; 3 Jiangsu Provincial Planning and Design Group, Nanjing 210023, China; huanglj@jspdg.com; 4 Graduate School of Engineering, Tohoku University, Sendai 980-0845, Japan; gao.jingyi.s4@dc.tohoku.ac.jp

**Keywords:** emergency management, public satisfaction, SEM, public health, COVID-19

## Abstract

Service satisfaction with public policies is an important component of public service quality management, which is of great significance to the improvement of public service quality. Based on an online questionnaire survey and in combination with the characteristics of public policies and services, in this study the influencing factors of residents’ satisfaction with COVID-19 pandemic prevention services were analyzed with structural equation modeling. The results reveal that the data fit the model well, and all the hypotheses formulated in this study were supported. Among the factors that were found to directly affect residents’ satisfaction with pandemic prevention services, perceived quality (PQ) has the greatest impact on satisfaction, followed by the disaster situation (DS) and policy expectation (PE). The observed variables that have significant impacts on the latent variables were also explored. Regarding the main findings, the residents who were seriously affected by the pandemic tended to have lower satisfaction with the policies and services provided by the government. Moreover, the improvement of PQ was found to significantly increase pandemic prevention service satisfaction (SS). Finally, the residents with a good psychological status during the pandemic were found to have higher satisfaction. According to the results, implications for the prevention and control practices of similar public health emergencies are proposed.

## 1. Introduction

At the end of 2019, the COVID-19 pandemic had quickly become a global threat to public health; by 1 May 2021, over 153 million people had been infected with the virus causing COVID-19, which devastatingly cost more than 3.2 million lives. Since its emergence, COVID-19 has been a leading cause of death worldwide, and has indirectly caused a considerable number of additional deaths [1]. In China, the COVID-19 pandemic has been a major public health emergency; since the founding of New China, it has been the fastest-spreading pandemic, the cause of the widest range of infections, and the most difficult to prevent and control [2]. The sudden pandemic has had a huge impact in the field of public health, and has caused instability to the normal operation of the social economy. To curb the development of the pandemic, governments worldwide have introduced various targeted policies, measures, and public services; these have met the actual needs of residents to a certain extent, and have played an important role in pandemic prevention. Moreover, in practice, the multi-faceted improvement of pandemic prevention policies and services has been a matter of continuous discussion. Experience and research have confirmed that public support, participation, and compliance are the keys to controlling the spread of public health incidents [3]. In an emergency, the public services provided by the government are the first to respond to crises, and the process of service is also a process of risk communication with the public to help managers improve services [4]. With the development of social networks and new media, the manner of decision-making for the public to participate in crisis events is becoming more diverse [5,6]. The advent of the big data era has provided the public with more opportunities to participate in crisis management and perceive the risk situation, and real-time information dissemination has changed the traditional decision-making model [7,8]. Therefore, against the current background of the highly convenient access to data and information, public satisfaction should be fully considered in the formulation and improvement of public policies [9,10,11]. In other words, public satisfaction builds a bridge of trust between the government and the people, which helps mitigate risks, especially regarding the current COVID-19 pandemic.

Satisfaction is generally conceptualized as an attitude similar to judgment following the act of purchasing or based on a series of consumer-product interactions, and the conceptualization and measurement of satisfaction are changing with societal development [12,13,14]. In the fields of economics and sociology, satisfaction is often used to measure employees’ satisfaction with their careers and customers’ satisfaction with products; rich research results have been obtained, especially in the exploration of customer satisfaction [15,16]. When contacting products and public services, the public has expectations that, together with the service process, affect their satisfaction [17]. Among the existing methods and models for the measurement of satisfaction, the American Customer Satisfaction Index (ACSI) is a widely used and highly recognized classical satisfaction model with a solid theoretical foundation, the purpose of which is to explore the customer’s satisfaction with a certain product based on a series of analyses of the relationships between variables. The model includes six latent variables, namely perceived quality, perceived value, customer expectations, customer satisfaction, customer complaints, and customer trust, as well as 14 observed variables. The ACIS is characterized by high quality in terms of both consistency reliability and test–retest reliability, and is often used to measure public satisfaction with public services [18,19,20,21]. In recent years, satisfaction measurement methods have been improved and innovated. For instance, Kar [22] used the sentiment mining and topic modeling methods to analyze Twitter data, and found that the factors of cost, usefulness, trust, social influence, credibility, information privacy, and responsiveness are more important for the improvement of user satisfaction with mobile payment services. Ilyas et al. [23] analyzed the role of brand awareness in the direct and indirect influences of the variables of customer satisfaction and buy-back willingness based on the online survey method; moreover, their data analysis and testing tools included the structural equation modeling (SEM) method using AMOS as the statistical data analysis software. Zhang et al. [21] considered the characteristics of Chinese public transport services, and modified the American customer satisfaction theory to construct a conceptual model of passenger satisfaction index; the model was then solved by partial least squares (PLS) regression and SEM to measure public transport passenger satisfaction. Sumiadji et al. [24] used the questionnaire method to explore the level of student satisfaction with the quality of service provided by their university’s accounting department; the questionnaire consisted of the five dimensions of tangibility, reliability, responsiveness, assurance, and empathy. Moreover, descriptive analysis was performed in terms of the mean and frequency, and the student satisfaction index was determined via importance-performance analysis.

Public service satisfaction refers to the public’s subjective perception and evaluation of the overall performance of a service in terms of the service process, service content, service attitude, and service effect during or after receiving government public services [25]. Under the influence of the new public management movement, public service satisfaction is becoming an important criterion for the evaluation of the level of government-provided public services. Public satisfaction evaluation has been applied to the public service evaluation of government departments, and numerous related studies have been conducted in fields including government performance evaluation, e-government service quality, urban management services, public medical services, and basic education services [26,27,28,29]. For example, Rhee and Rha [30] developed an alternative model of public service quality via the critical incident technique in consideration of four main qualities of public service, namely the process quality, outcome quality, design quality, and relationship quality; the results revealed that the key attributes of customer satisfaction with public service quality vary with the types of public sector customers. Kampen et al. [31] studied how satisfaction with service delivery affects trust in public institutions in an empirical environment, and found that the impact on the negative experience of public institutions is much more pronounced than the impact on the positive experience; therefore, reducing the number of disappointed people has a greater impact on increasing trust in the public sector than increasing the number of pleased people. Mbassi et al. [32] conducted a questionnaire survey of 1427 local public service users from 21 councils, and then evaluated the relationship between the public service quality of local municipalities and citizen-client satisfaction based on the revisited service quality (SERVQUAL) model. Wu [33] used data from a citizen survey of the City and County of San Francisco to test the relationship between citizens using the “311 system” to contact City Hall, as well as citizen satisfaction with the quality of public service encounters. To weigh in on the controversy among scholars regarding how rewards impact the relationship between public service motivation and job satisfaction, Li et al. [34] conducted a public product game experiment among 195 college students, and task satisfaction was measured as an agent of job satisfaction. The purpose of exploring public service satisfaction and its influencing factors is to improve service quality and increase trust between the government and the public. With the development of multi-source big data, satisfaction measurement methods, such as those based on big data and text mining, have become diversified, but interviews and questionnaires are often still used in conjunction with these methods [35,36,37,38].

The speed of the spread and recovery of the COVID-19 pandemic is closely related to prevention and control measures, which, to a certain extent, affect the public’s psychological recognition of pandemic prevention services [39,40,41]. Some studies have measured the satisfaction level of patients and medical staff during the COVID-19 period. For example, Yu et al. [42] used a questionnaire to measure the job satisfaction of medical staff in China’s fight against COVID-19, which has important reference value for the improvement of the job satisfaction level of frontline medical staff in public health emergencies, the formulation of medical staff safety guarantee policies, and the promotion of the construction of emergency teams. Deriba et al. [43] analyzed data collected based on a structured interviewer-administered questionnaire in North Shoa healthcare facilities, and found that patient satisfaction was very low during the COVID-19 pandemic. Moreover, the presence of sign and direction indicators, the availability of drugs, social distancing, the availability of alcohol, and the availability of sanitizer were factors found to be associated with patient satisfaction. An assessment in Tigray, Ethiopia, showed that although medical staff have sufficient knowledge of COVID-19, their cognition and job satisfaction are still concerns of the healthcare system; to control COVID-19, efforts should be exerted to improve perceptions, job satisfaction, and readiness [44]. To understand public satisfaction with public transportation after the COVID-19 pandemic, Dong et al. [45] conducted a cross-sectional survey of eight Chinese cities where public transportation systems were temporarily closed due to the pandemic, and suggestions were proposed to avoid the loss of ridership. Regarding the perception of the telework/tele-study space in Mexico, a study by Jaimes Torres et al. [46] found that, in general, households were satisfied with the size of their houses, but would like landscaped spaces or better outdoor views; thus, new architectural design paradigms must be rethought. Some other related research perspectives have also been explored during the COVID-19 pandemic, such as life satisfaction [47], customer satisfaction [48], student satisfaction [49], and tourist satisfaction [50].

Most current studies on satisfaction are aimed at the measurement of satisfaction with actual currency and commodity exchange processes. Regarding the studies on public service, public service is mainly used as the evaluation object, and there have been few studies on the influence and mechanism of the relationship between public service and policy satisfaction. Based on the ACSI, the impact effects of perceived quality, policy expectation, resident complaints, resident trust, and disaster situation on pandemic prevention service satisfaction were explored, and six hypotheses were proposed in the present work: (1) disaster situation has a significant positive impact on policy expectation; (2) disaster situation has a significant negative impact on pandemic prevention service satisfaction; (3) policy expectation has a significant negative impact on pandemic prevention service satisfaction; (4) perceived quality has a significant positive impact on pandemic prevention service satisfaction; (5) pandemic prevention service satisfaction has a significant negative impact on resident complaints; (6) pandemic prevention service satisfaction has a significant positive impact on resident trust. We collected 516 effective responses from an online survey conducted in Nanjing, China. Furthermore, according to the preliminary analysis of the questionnaire data, six latent variables and 34 observed variables were used to construct a structural equation model to measure residents’ satisfaction with COVID-19 pandemic prevention services and its influencing factors. The internal relationships between public satisfaction and the observed and latent variables were explored with the goal of providing a reference for the improvement of public service satisfaction. Moreover, the implications of the policy response to similar emergencies are put forward. It is the hope of the authors that this work improves the quality of public policies and public satisfaction against the background of the current COVID-19 pandemic.

## 2. Materials and Methods

### 2.1. Theoretical Hypotheses

**Figure 1:**
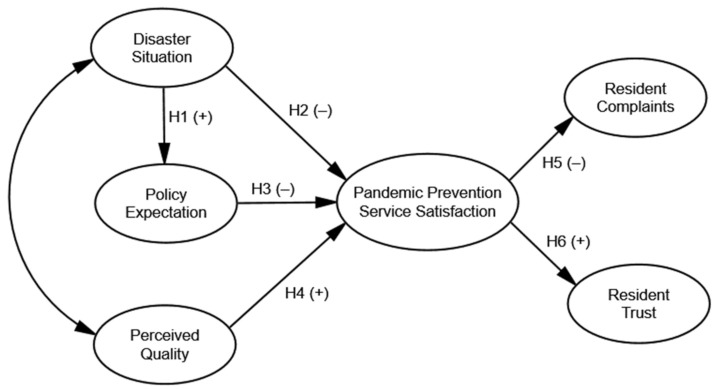
The theoretical framework.

According to the ACSI, customer expectations affect perceived quality, perceived value, and customer satisfaction. Moreover, perceived quality has an impact on perceived value and customer satisfaction. Customer satisfaction affects customer behavior, including the loyalty and complaints of customers [18]. In this study, the specific content of the ASCI was adopted to retain the latent variable of perceived quality (PQ), while customer expectation, customer satisfaction, customer complaints, and customer loyalty were derived into the corresponding latent variables of policy expectation (PE), pandemic prevention service satisfaction (SS), resident complaints (RC), and resident trust (RT). Compared with the ACSI, based on practical considerations, the main changes of the structural equation model constructed in this study were the addition of the latent variable of the disaster situation (DS), and the removal of the latent variable of perceived value. Figure 1 presents the hypotheses and the conceptual framework.

Residents may be directly infected during the pandemic, or indirectly affected in life and work. When in a public emergency, people will seek help from the government. When the government helps people get rid of the predicament, they will recognize the public policies provided by the government and have higher expectations for public policies. Similarly, when people feel that they have been severely affected, they will show a negative attitude towards the current pandemic control measures and services. Therefore, the following hypotheses were proposed.

**Hypothesis 1** **(H1):** *Disaster situation has a significant positive impact on policy expectation.*

**Hypothesis 2** **(H2):** *Disaster situation has a significant negative impact on pandemic prevention service satisfaction.*

As known, people always have expectations for public services. For COVID-19, pandemic prevention services mainly come from the governments, NGOs, and volunteers. The services have reduced the risks that individuals need to face to a certain extent. Due to the limited public resources, policies and services cannot maximize the satisfaction of residents’ expectations. When the public has high expectations for pandemic prevention services and their needs cannot be fully considered, their satisfaction with the services will be at a low level. Therefore, the following hypothesis was proposed.

**Hypothesis 3** **(H3):** *Policy expectation has a significant negative impact on pandemic prevention service satisfaction.*

ACSI had shown that there was a correlation between perceived quality and satisfaction. Perceived quality represents the public’s perception of COVID-19 pandemic, including the infectivity and spread of the virus, the risk areas, understanding of policies, and the identification of rumors. When individuals have a comprehensive and correct perception of the pandemic, they may be satisfied with the pandemic prevention services. Therefore, the following hypothesis was proposed.

**Hypothesis 4** **(H4):** *Perceived quality has a significant positive impact on pandemic prevention service satisfaction.*

As mentioned above, whether the government can help the public out of the plight of the pandemic will change their attitude. When residents are in a state of satisfaction with the pandemic prevention services, it indicates that they have been affected little and the control measures adopted by the government have alleviated the pandemic risk. Furthermore, residents tend not to complain about pandemic prevention services, and they may trust the government’s actions more. When there is good risk communication between the public and the government, pandemic prevention and control will be more effective. Therefore, the following hypotheses were proposed.

**Hypothesis 5** **(H5):** *Pandemic prevention service satisfaction has a significant negative impact on resident complaints.*

**Hypothesis 6** **(H6):** *Pandemic prevention service satisfaction has a significant positive impact on resident trust.*

### 2.2. Methods

#### 2.2.1. Variables and Structural Equation Modeling

The measured items for all the latent variables in this study were adopted and modified from the ACSI [19,20]. The specific contents of the latent variables are as follows: (1) Perceived quality (PQ) refers to the quality of the various services provided by the government perceived by the public after the outbreak of a pandemic, including the public perception of the authority of information channels provided by the government, the practicability of information, and the comprehensiveness of services. (2) Policy expectation (PE) generally refers to the public expectation of meeting the needs of the public regarding product quality and services before receiving services; the expectation is influenced by previous use experience and early product publicity. In this study, PE mainly refers to the comprehensive expectation of whether the government releases information and policies regarding COVID-19 in an accurate and timely manner. It also includes the expectation of the present interaction between the public and the government, as well as the expectation of the public to satisfy their own different service needs. (3) Pandemic prevention service satisfaction (SS) mainly refers to the public’s satisfaction with government service behavior after the pandemic. It is not only an important embodiment of the satisfaction of public needs, but also an important manifestation of the completion of government work. (4) Resident complaints (RC) refer to public dissatisfaction with and censure of the work and service of the government during the COVID-19 pandemic, and are mainly expressed by the opinions about the government and the discussion of inappropriate actions. (5) Resident trust (RT) refers to the trust in government institutions, government behavior, government decision-making, and the government system based on psychological expectation, emotion, and rational judgment; in this study, it mainly refers to the recognition and trust in government work and services during the pandemic, primarily including the good reputation of government services. (6) The disaster situation (DS) refers to the status of residents affected by the COVID-19 pandemic, and mainly includes the impact on daily life, work, study, and psychological and spiritual conditions. All the latent variables and observed variables are listed in Table 1.

**Figure 2:**
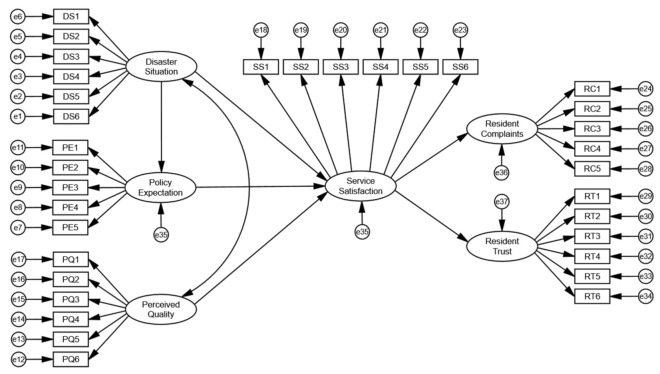
The construction of the structural equation model.

SEM is a multivariate statistical analysis technique that is used to analyze structural relationships. It involves the construction of a model and an informative representation of some observable or theoretical phenomenon. In SEM, different aspects of a phenomenon are theorized to be related to one another with a structure. The two basic models used in SEM are the measurement and structural models. The measurement model can reflect the relationship between latent and observed variables, while the structural model reflects the hypothesized causal relationship between latent variables. In the measurement of public service satisfaction, the influencing factors are latent variables, which cannot be directly measured; they must be indirectly measured by setting measurement indicators. After conducting a confirmatory factor analysis and adjusting the observation variables, a structural equation model of public satisfaction with COVID-19 prevention services was constructed, and included 34 observed variables and six latent variables (Figure 2).

#### 2.2.2. Data Collection and Processing

The questionnaire consisted of two parts, the first of which included the basic information of the participants, including their gender, age, education, and monthly income. The second part surveyed participants from dimensions of perceived quality, policy expectation, pandemic prevention service satisfaction, resident complaints, resident trust, and disaster situation, with item numbers of 6, 5, 6, 5, 6, and 6, respectively. According to the latent variables in the model, the problem items in the second part of the questionnaire were designed for the corresponding observed variables. The Likert five-point scale was adopted for the design of the measured questions; the possible responses were “very satisfied,” “satisfied,” “neutral,” “dissatisfied,” and “very dissatisfied,” with corresponding scores of 5, 4, 3, 2, and 1. Taking Nanjing residents as the objects to conduct the survey of the satisfaction with COVID-19 pandemic prevention services, “Wenjuanxing” (https://www.wjx.cn/) (assessed on 25 July 2020), an online survey platform, was used to distribute and retrieve questionnaires in July 2020.

IBM SPSS Statistics 24.0 was used to establish databases and perform statistical analyses. First, check the integrity and accuracy in order to screen out effective questionnaires. As known, reliability and validity tests are crucial for the subsequent analysis. Reliability refers to the repeated measurement of an object with the same method; it can not only indicate the consistency of the results, but can also explain the degree of reflection of the questionnaire to the actual situation. In this study, the internal consistency reliability test was used to judge whether the data were reliable based on the Cronbach’s α coefficient, the criteria of which are as follows: if the reliability of the whole scale is between 0.7–0.8, it indicates that the reliability is acceptable; if the reliability of the subscale is between 0.6–0.7, it indicates that the reliability is acceptable. Validity represents whether the questionnaire can effectively reflect the contents to be examined; the higher the validity, the more consistency between the measurement results and the contents examined. The convergence validity of the samples was evaluated mainly by exploratory factor analysis (EFA) and confirmatory factor analysis (CFA). EFA is to measure the structural validity of the scale and to judge whether the observed variables of each latent variable have a stable consistency and structure, and the purpose of CFA is to test whether the relationship between a factor and the corresponding measurement item conforms to the designed theoretical relationship. 

## 3. Results

### 3.1. Effective Questionnaire Screening and Descriptive Statistics

The data were pretreated from two aspects of integrity and accuracy check. Data integrity is mainly to check whether the items of the questionnaire are completed and whether there are any omissions. The accuracy check is to find out whether the survey data can truly reflect the actual situation and whether the data are wrong; 516 valid questionnaires were finally obtained. The sample included 281 men (54.46%) and 235 women (45.54%), with a relatively balanced gender ratio. Most of the participants were in the range of 31 to 45 years old (33.53%). The proportion of respondents whose educational experience was junior college 33.92%, followed by high school, secondary school, and vocational high school (24.03%). The monthly income of most respondents was between 3000–5999 (RMB). The specific participant statistics are reported in Table 2.

### 3.2. Reliability and Validity Testing

The reliability test results showed that the Cronbach’s α values of all latent variables were at an acceptable level, ranging from 0.851 to 0.899 (>0.7), and the CITC (corrected item-total correlation) between the observed variable and latent variable met the requirement of greater than 0.5 (Table 3), indicating that the questionnaire had a high reliability. Additionally, excluded observations on the observed variables were performed. If the reliability index does not increase after the variable is deleted, it is considered that the measurement item of the variable has a good reliability. The test showed that the overall Cronbach’s α coefficient was not improved after deleting each item, indicating that the item setting was appropriate.

The use of exploratory factor analysis (EFA) for the validity test generally needs to meet two conditions: the KMO value is greater than 0.7; the significance of Bartlett’s test of sphericity is less than 0.05. The KMO value of the survey data was 0.909, and the result of Bartlett’s test of sphericity showed that the approximate chi-square value was 9076.504, and the null hypothesis of Bartlett’s test of sphericity was rejected. The scale was suitable for factor analysis, and its validity was well structured. In the EFA process, principal factor analysis was used to extract six common factors with eigenvalues greater than 1, and it was found that the total variance explanation rate of the six factors was 65.02% (>60%). The 34 question items were classified into six types of factors by the maximum variance method, and the load of each measurement item was higher than 0.5 (Table A1). There was no high dual factor load, and the measurement items in each dimension were aggregated according to the theoretical distribution, indicating that the questionnaire had a good content validity.

**Figure A1:**
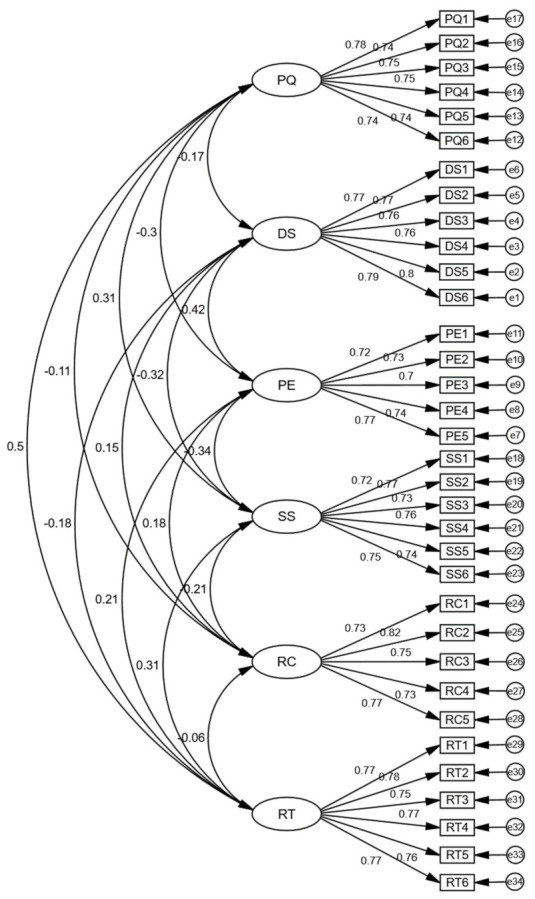
Parameter estimates (standardized coefficients).

In confirmatory factor analysis (CFA), the overall fit of the CFA model (Figure A1) was firstly analyzed according to the fitness indicators most frequently reported in previous studies [51], including χ^2^ (chi-square), df (degree of freedom), χ^2^/df, GFI (the goodness of fit index), CFI (the comparative fit index), TLI (the Tucker–Lewis index), and RMSEA (the root-mean-square error of approximation), with values of 734.261, 512, 1.434, 0.912, 0.975, 0.972, and 0.029, respectively. There is a certain reasonable value range for each of these indicators. Among them, the recommended RMSEA is less than 0.1, which is acceptable, and less than 0.05 is ideal [52]; the recommended GFI is more than 0.9 [53]; the recommended CFI is more than 0.9 [54]; the recommended χ^2^/df is between 1 and 3, and it is within the acceptable range if not more than 5; finally, the recommended TLI is above 0.9 [55]. Therefore, the CFA model was found to exhibit a good fit with the survey data. Next, based on the CFA, composite reliability (CR) and average variance extracted (AVE) were tested. The results showed that the standard factor load of each item was significant, which met the requirement of a recommended value of 0.6 or more; the CR of each latent variable was between 0.852 and 0.9, and the CR is considered acceptable if the value is above 0.7 [56]; the average of variance extracted (AVE) of each latent variable was greater than 0.5, indicating that the scale had a good convergence validity [57] (Table 3). In summary, the scale in this study passed the reliability and validity test.

### 3.3. Hypothesis Testing and Impact Effect between the Factors

First, whether there was a good fit between the SEM and the survey data was analyzed. According to the chi-squared test, the value of χ^2^/df was 1.637, which was statistically significant, where the smaller the value of χ^2^/df, the better the goodness of fit. The GFI (the goodness of fit index) was 0.901, which should be lower than 1. The RMSEA (the root-mean-square error of approximation) had a value of 0.035 (<0.1). These indexes indicated a good fit. The coefficients between latent variables indicate the degree of variation of the other variables caused by the variation of one variable. The analysis of path coefficients between the latent variables for pandemic prevention service satisfaction are shown in Table 4. The results revealed that DS had a significant positive impact on PE, DS had a significant negative impact on SS, PE had a significant negative impact on SS, PQ had a significant positive impact on SS, SS had a significant negative impact on RC, and SS had a significant positive impact on RT. All the hypotheses were supported.

**Figure 3:**
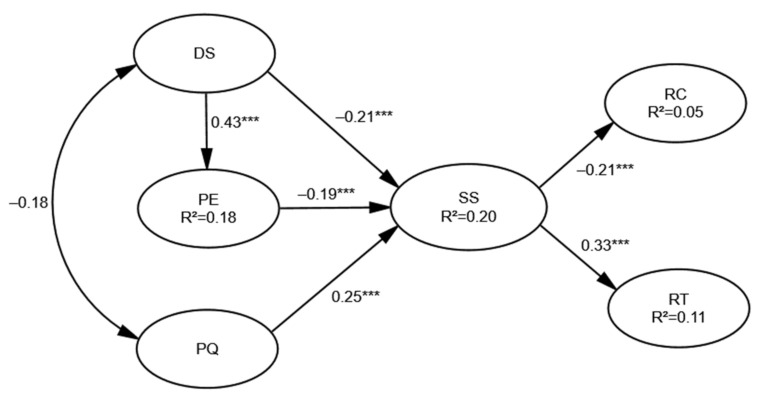
Path estimates of the model (*** *p* < 0.001).

The impact effects between the latent variables were analyzed. The results showed that PQ, PE, DS, RC, and RT were all found to have an impact on SS, among which only the DS was found to have both direct and indirect impacts. Moreover, PQ and PE were found to have direct impacts on SS, and SS was found to have a direct impact on RC and RT. The relationships between the latent variables are presented in Figure 3. This study mainly focused on the analysis of the relationship between pandemic prevention service satisfaction and other variables. The correlation analysis of the various factors is as follows: (1) for hypothesis H2, DS5 had the greatest impact on this dimension with a loading coefficient of 0.80, indicating that strengthening the guidance of individual psychological state was crucial for improving pandemic prevention service satisfaction, followed by DS6, DS1, and DS2. (2) Among the observed variables related to hypothesis H3, the loading coefficients of PE5 and PE4 were 0.76 and 0.74, which revealed that enhancing the professionalism of the measures and services adopted by the government in the pandemic prevention and control, as well as the action capacity, were helpful to improving the satisfaction of residents. (3) For hypothesis H4, PQ1 had a highest loading coefficient of 0.77, indicating that whether the public was aware of the way the virus spreads was very important to their satisfaction. (4) In view of the results that SS had different impacts on RC and RT (H5, H6), we should first improve the effectiveness of the pandemic response policies (SS2, 0.77) and the professionalism of the policies (SS4, 0.75), which had great impacts on the satisfaction. On the other hand, RC2 (0.82), RC5 (0.77), RT2 (0.78), RT1 (0.77), RT4 (0.77), and RT6 (0.77) should be given enough attention, indicating that reducing complaints, and increasing appreciation and trust in pandemic prevention policies were also crucial for improving public satisfaction.

## 4. Discussion and Limitations

In public health emergencies, the disaster situation tends to have a direct effect on RC of SS in the form of PE. Regarding the latent variable paths, the path coefficient of the DS to SS was −0.206, which passed the *t*-value test; this indicated that the extent to which the residents were affected by the COVID-19 pandemic directly affected their satisfaction with pandemic prevention services. The more severely affected residents were, the lower their satisfaction with the services. Among the five observed variables of PE, “My expectation of the professionalism of the pandemic response policies (PE5)” and “My expectation of the action capacity of the relevant departments (PE4)” were found to have great impacts, the factor loadings of which reached more than 0.74, indicating that the residents had high expectations for policy professionalism and action capacity. The path coefficient of the PE to SS was −0.19, which showed that PE significantly and negatively affected SS. The DS was found to have an indirect impact on SS by influencing PE, with a path coefficient of 0.43, and passed the *t*-value test. The preceding analysis demonstrates that the DS affected the residents’ satisfaction with COVID-19 prevention services by affecting their PE, i.e., compared with the residents less affected by the pandemic, those residents who were seriously affected by the pandemic had greater expectations for the pandemic prevention policies and services provided by the government, and their satisfaction with pandemic prevention services was lower, which is also in line with the general relationship between expectation and satisfaction, that is, expectation and satisfaction are negatively correlated. When the expectation value tends to infinity, satisfaction will also approach zero. Considering that the expectation of public services is a subjective judgment on future public services based on the perception of past or existing government public services, the results prove that the actual service quality of COVID-19 pandemic prevention in Nanjing was higher than the public expectation. Furthermore, they also indicate that the addition of the latent variable of the DS to the evaluation model constructed in this study was practicable.

It was found that the improvement of PQ can induce a significant increase in residents’ satisfaction with pandemic prevention services, and the path coefficient of PQ to SS reached 0.246 and passed the *t*-value test, indicating that PQ positively affected SS. Via the in-depth analysis of the six observed variables of PQ, it was found that “I understand how COVID-19 spreads (PQ1)”, “I understand the infectiousness of COVID-19 (PQ2)”, “I can identify which type of mask is suitable for preventing COVID-19 (PQ3)” and “I understand the number of infected people and the distribution of the hardest-hit areas (PQ4)” had obvious impacts on PQ, the factor loadings of which all exceeded 0.75. This indicates that the services provided by the government departments related to COVID-19 prevention during the pandemic period (including the active publicity and popularization of relevant pandemic information, informing the public how to protect against the pandemic, announcing the pandemic situation in various areas, etc.) imparted the residents with a sense of the reliability, stability, and security of public services, the professionalism of staff, etc. On the other hand, the residents also realized the integrity, timeliness, and practicability of the information provided by the government, thereby providing the public with a greater sense of security during the pandemic and ultimately promoting the improvement of SS.

Numerous studies in the field of sociology have focused on human psychology, and the same is true for public health emergencies. The analysis of the impact effects between variables demonstrated that the psychological state of residents indirectly had a significant positive impact on the satisfaction with pandemic prevention services. For the latent variable DS, the influence of the observed variable of “The extent to which my psychological state has been affected by the pandemic (DS5)” was found to be significant, and the factor loadings reached more than 0.80, indicating that the residents with good psychological status during the pandemic had higher satisfaction with COVID-19 prevention services; this may have been caused by the differences between the original public lifestyle and the lifestyle after the outbreak of the pandemic. In this process, the public may have been fully prepared for physical precautions, but many people may not have been able to take psychological precautions. The huge pressure imposed during the pandemic resulted in a variety of psychological problems in the public. In addition, during the pandemic, the public could rarely interact with the outside world, which may have led to negative emotions such as anger and anxiety. When individuals are dominated by a variety of negative emotions, they tend to close their cognition, and simply rely on existing standards to make judgments; during COVID-19, they used these judgments to evaluate the pandemic prevention services, which ultimately affected their satisfaction with these services.

This study provides a constructive reference for government departments to provide high-quality services from the perspective of public needs, especially for the current COVID-19 pandemic. However, some limitations require further exploration. Generally, public service satisfaction involves complex stakeholders and social environments, and is an issue that exists against a specific socioeconomic background. In public management, when a public service or product is launched by a government, there usually exists a multi-party game process. At this time, the government, the public, and third-party organizations will put forward different demands based on their own interests, which will have a direct impact on the experience of receiving services. Therefore, when designing a questionnaire, the demands of multiple parties should be considered as much as possible to obtain more inclusive results. Moreover, the level of economic development of different regions is different, as is the ability of governments to provide public services, which may have a direct impact on public satisfaction. From the human perspective, the needs of vulnerable groups in many emergencies are very important, including those of the elderly, children, and people with reduced mobility. These groups have specific needs, and in consideration of the economy, maximum coverage, etc., these needs are often not fully met when formulating public services. In addition, because the COVID-19 pandemic in some cities and provinces is in a recurring stage and the variants of the virus appeared in some regions, public services for pandemic prevention will undergo frequent changes, which may have an impact on public psychology and affect satisfaction with pandemic prevention services. Therefore, the findings of this study have limited practical applications for the current pandemic prevention and control, and more exploration needs to be carried out in combination with the current background and future trends. By adding more dimensions to the questionnaire design, interviews and surveys on different subjects can be made more practicable.

## 5. Conclusions and Implications

### 5.1. Conclusions

The investigation of the satisfaction with pandemic prevention services helps to achieve trustworthy risk communication between the public and the government, thereby helping to control the spread of the pandemic. In this study, an online survey was conducted in July 2020, and 516 valid responses were obtained. The structural equation model constructed in this work included the six latent variables of PQ, PE, SS, RC, RT, and DS, along with 34 observed variables. The Cronbach’s α values of all latent variables were at an acceptable level, ranging from 0.851 to 0.899 (>0.7), indicating that the scale had a high reliability, and the results of EFA and CFA showed a good validity of the survey data. According to the analysis of path coefficients between the latent variables, the coefficients of DS to PE, DS to SS, PE to SS, PQ to SS, SS to RC, and SS to RT were 0.427 ***, −0.206 ***, −0.193 ***, 0.246 ***, −0.213 ***, and 0.325 ***, respectively, revealing that all the hypotheses put forward were supported. The impact effects between the latent variables revealed the following: PQ, PE, DS, RC, and RT were all found to have an impact on SS, among which only the DS was found to have both direct and indirect impacts on SS; PQ and PE were found to have direct impacts on SS; SS was found to have direct impacts on RC and RT. Furthermore, the three main findings of this study were as follows: (1) The DS was found to affect SS by affecting PE, i.e., compared with the residents less affected by the pandemic, those residents who were seriously affected had greater expectations of the policies and services provided by the government, and their satisfaction with pandemic prevention services was lower. (2) The improvement of PQ can cause a significant increase in SS, and the observed variables of PQ1, PQ2, PQ3, and PQ4 were found to have obvious impacts on PQ; this indicates that the services provided by the government departments related to COVID-19 prevention can impart the public with the feelings of reliability, stability, and security regarding public services, and can allow the public to realize the integrity, timeliness, and practicability of the information. (3) Regarding the DS, the observed variable of “The extent to which my psychological state has been affected by the pandemic” was found to have a significant influence on SS with a factor loading of 0.80, indicating that the residents with good psychological status during the pandemic tended to have higher satisfaction with COVID-19 prevention services.

As mentioned in the discussion section, public satisfaction involves multiple stakeholders, and there are many influencing factors. This research took Nanjing as the study object, and a questionnaire survey on residents’ satisfaction with COVID-19 pandemic prevention was conducted. To reduce the problem items to facilitate data acquisition, simple processing was carried out in multiple dimensions. In addition, the conclusions have certain limitations. Nevertheless, much more related research should be conducted in the future. First, the impact mechanisms of the related variables should be further explored from more dimensions, and comparative analyses of factors in other regions should be conducted. Second, considering the interests of multiple parties, to discover how the influencing factors change over time, questionnaires and interviews are necessary. Third, based on the general applicability of the results, it is necessary to strengthen the surveys of public health service satisfaction by vulnerable groups. Finally, with the widespread application of big data, the methods of satisfaction research should be innovated based on multi-source data to yield more objective results.

### 5.2. Practical Implications

Public policies are always in progress. Whether considered from the perspectives of the public, the government, or third-party organizations, the experience and lessons learned from emergencies handling will be summarized to respond more successfully and adequately in the future. According to the research results, the following suggestions are put forward for the responses to similar public health emergency events.

(1) The professionalism of pandemic prevention policies and the action capacity of the relevant departments should be enhanced. Professional knowledge represents professionalism; thus, policies can be made feasible by being supported by sufficient expertise. In similar public health incidents, doctors, researchers, and scholars in related fields are not only personnel with sufficient professional knowledge, but are also individuals trusted by the public. In the process of the government issuing relevant policies and response measures, personnel with sufficient professional knowledge and professional discourse power should conduct supervision, guidance, and policy evaluation. The action capacity shows the efficiency of policy formulation and the speed of action by relevant departments. A strong action capacity can make the public have confidence in epidemic prevention and trust in the pandemic prevention services provided by the government.
(2) The propaganda and popularization of relevant basic knowledge of pandemic prevention and basic pandemic information should be strengthened. The main PQ factors affecting residents’ satisfaction with pandemic prevention services were found to be “I understand how COVID-19 spreads,” “I understand the infectiousness of COVID-19,” “I can identify which type of mask is suitable for preventing COVID-19,” and “I understand the number of infected people and the distribution of the hardest-hit areas”; in view of this, when similar public health incidents occur, focus should be placed on strengthening the publicity and popularization of the infectivity of the disease, relevant protective measures, and the distribution of the hardest-hit areas. The publicity of various types of basic pandemic prevention information should be detailed to communities and residential areas, and community WeChat groups, official accounts, and SMS point-to-point distribution must be fully utilized; this will allow residents to learn about pandemic-related information in a timely and convenient manner.
(3) Attention should be paid to the changes in the psychological state of residents during the pandemic, and psychological counseling for the public should be strengthened. Considering that “The extent to which my psychological state has been affected by the pandemic” was found to significantly affect residents’ satisfaction with pandemic prevention, the changes in residents’ psychological state during a pandemic period deserve more attention. At present, most of the psychological counseling work for residents during the pandemic period has been via text or video chat, which has not played an effective role. The community must gradually establish a complete psychological counseling service process and establish a psychological counseling service station. Personnel with professional psychology knowledge can provide community residents with a face-to-face communication platform to protect residents’ psychological safety during a pandemic.

## Figures and Tables

**Table 1:** The latent and observed variables.

Latent Variable	Observed Variable
Perceived quality (PQ)	(PQ1). I understand how COVID-19 spreads
	(PQ2). I understand the infectiousness of COVID-19
	(PQ3). I can identify which type of mask is suitable for preventing COVID-19
	(PQ4). I understand the number of infected people and the distribution of the hardest-hit areas
	(PQ5). I can correctly identify numerous rumors about the pandemic
	(PQ6). I understand the pandemic prevention policies
Policy expectation (PE)	(PE1). My expectation of the response speed of relevant departments
	(PE2). My expectation of the effectiveness of the pandemic response policies
	(PE3). My expectation of the feasibility of the pandemic response policies
	(PE4). My expectation of the action capacity of the relevant departments
	(PE5). My expectation of the professionalism of the pandemic response policies
Pandemic prevention service satisfaction (SS)	(SS1). My satisfaction with the action capacity of the pandemic response policies
	(SS2). My satisfaction with the effectiveness of the pandemic response policies
	(SS3). My satisfaction with the feasibility of the pandemic response policies
	(SS4). My satisfaction with the professionalism of the pandemic response policies
	(SS5). My satisfaction with the acquisition of the pandemic prevention materials
	(SS6). My overall satisfaction
Resident complaints (RC)	(RC1). I believe there is a tendency to complain about pandemic prevention services
	(RC2). I complain about pandemic prevention services to acquaintances
	(RC3). I complain about pandemic prevention services on social media
	(RC4). I express dissatisfaction with pandemic prevention services to relevant departments
	(RC5). An acquaintance has complained to me about pandemic prevention services
Resident trust (RT)	(RT1). I tend to praise the government’s pandemic prevention work.
	(RT2). I praised the pandemic prevention services to my friends.
	(RT3). I praise the pandemic prevention services on social media and the Internet
	(RT4). I trust the pandemic prevention information provided by the governments
	(RT5). I believe that the risk of infectious diseases will become higher and higher in the future
	(RT6). I will continue to support the work of relevant departments in the future
Disaster situation (DS)	(DS1). The extent to which my daily life has been affected by the pandemic
	(DS2). The extent to which my work has been affected by the pandemic
	(DS3). The extent to which my social interaction has been affected by the pandemic
	(DS4). The extent to which my health has been affected by the pandemic
	(DS5). The extent to which my psychological state has been affected by the pandemic
	(DS6). The extent to which my family and friends have been affected by the pandemic

**Table 2:** The sample demographics.

Characteristic	Range	Frequency	Percentage
Gender	Male	281	54.46
	Female	235	45.54
Age	≤17	61	11.82
	18–30	149	28.88
	31–45	173	33.53
	46–60	82	15.89
	≥61	51	9.88
Education	Primary school and below	31	6.01
	Junior high school	59	11.43
	High school, secondary school, and vocational high school	124	24.03
	Junior college	175	33.92
	Bachelor’s degree	97	18.80
	Master’s degree and above	30	5.81
Monthly income (RMB)	<3000	136	26.36
	3000–5999	206	39.92
	6000–9999	115	22.29
	≥10,000	57	11.04
	None	2	0.39

**Table 3:** The main information from exploratory factor analysis (EFA) and confirmatory factor analysis (CFA).

Latent Variable	Observed Variable	Standard Load	CITC	T	*p*	CR	AVE	Cronbach’s α
PE	PE1	0.719	0.654			0.852	0.535	0.851
	PE2	0.725	0.653	14.954	***			
	PE3	0.704	0.644	14.548	***			
	PE4	0.741	0.673	15.269	***			
	PE5	0.766	0.688	15.708	***			
PQ	PQ1	0.778	0.719			0.885	0.562	0.885
	PQ2	0.742	0.696	17.181	***			
	PQ3	0.751	0.692	17.415	***			
	PQ4	0.745	0.695	17.268	***			
	PQ5	0.741	0.69	17.173	***			
	PQ6	0.739	0.689	17.113	***			
RC	RC1	0.728	0.668			0.872	0.578	0.872
	RC2	0.821	0.751	17.364	***			
	RC3	0.748	0.69	15.944	***			
	RC4	0.734	0.677	15.66	***			
	RC5	0.766	0.706	16.313	***			
RT	RT1	0.765	0.717			0.895	0.588	0.895
	RT2	0.783	0.727	18.149	***			
	RT3	0.751	0.706	17.314	***			
	RT4	0.772	0.723	17.845	***			
	RT5	0.757	0.713	17.456	***			
	RT6	0.771	0.722	17.838	***			
SS	SS1	0.722	0.675			0.881	0.552	0.881
	SS2	0.769	0.711	16.392	***			
	SS3	0.726	0.673	15.493	***			
	SS4	0.756	0.703	16.128	***			
	SS5	0.738	0.678	15.763	***			
	SS6	0.745	0.694	15.895	***			
DS	DS1	0.766	0.72			0.9	0.6	0.899
	DS2	0.770	0.723	17.876	***			
	DS3	0.760	0.712	17.617	***			
	DS4	0.764	0.724	17.726	***			
	DS5	0.796	0.741	18.559	***			
	DS6	0.788	0.74	18.351	***			

*Notes:* “***” indicates significance at the 0.001 level.

**Table 4:** The path coefficient between the latent variables.

Path Relation	Standardized Estimate	Standard Error	T Statistics	*p*
Disaster situation → Policy expectation (H1)	0.427	0.041	8.301	***
Disaster situation → Pandemic prevention service satisfaction (H2)	−0.206	0.039	−3.834	***
Policy expectation → Pandemic prevention service satisfaction (H3)	−0.193	0.048	−3.57	***
Perceived quality → Pandemic prevention service satisfaction (H4)	0.246	0.043	5.029	***
Pandemic prevention service satisfaction → Resident complaints (H5)	−0.213	0.069	−4.191	***
Pandemic prevention service satisfaction → Resident trust (H6)	0.325	0.063	6.379	***

*Notes:* “***” indicates significance at the 0.001 level.

## Appendix A

**Table A1:** The main information of exploratory factor analysis.

Observed Variable	1	2	3	4	5	6
PQ1	0.774					
PQ2	0.784					
PQ3	0.745					
PQ4	0.768					
PQ5	0.757					
PQ6	0.761					
DS1		0.794				
DS2		0.789				
DS3		0.77				
DS4		0.814				
DS5		0.786				
DS6		0.81				
PE1			0.753			
PE2			0.734			
PE3			0.762			
PE4			0.778			
PE5			0.773			
SS1				0.77		
SS2				0.778		
SS3				0.742		
SS4				0.783		
SS5				0.741		
SS6				0.775		
RC1					0.795	
RC2					0.841	
RC3					0.796	
RC4					0.786	
RC5					0.811	
RT1						0.779
RT2						0.765
RT3						0.78
RT4						0.783
RT5						0.794
RT6						0.788
Eigenvalue	3.126	7.978	2.013	2.609	2.328	4.052
Variance contribution rate	9.19%	23.46%	5.92%	7.67%	6.85%	11.92%
Total variance contribution rate	65.02%					
